# Research on the Applicability of Touchscreens in Manned/Unmanned Aerial Vehicle Cooperative Missions

**DOI:** 10.3390/s22218435

**Published:** 2022-11-02

**Authors:** Hongjun Xue, Qingpeng Zhang, Xiaoyan Zhang

**Affiliations:** 1 School of Aeronautics, Northwestern Polytechnical University, Xi’an 710072, China; 2 School of Marine Science and Technology, Northwestern Polytechnical University, Xi’an 710072, China

**Keywords:** manned/unmanned, perceived workload, touchscreen, time pressure, task difficulty

## Abstract

The suitability of touchscreens for human–computer interaction in manned/unmanned aerial vehicle cooperative missions remains uncertain, especially in situations that are time-sensitive with variations in difficulty levels. The purpose of this study is to determine the feasibility of touchscreen applications in manned/unmanned aerial vehicle cooperative missions and the magnitude of the effects of time pressure and task difficulty. In contrast to previous studies, a combination of performance and perceptual load measures was used to divide errors into disposition errors, undetected errors, and miscalculation errors to explore specific error mechanisms, set up typical manned/unmanned aerial vehicle cooperative human–computer interaction tasks, and set up antecedent features for potential factors. Thirty subjects participated in an experiment that required the use of touchscreens or keyboards to perform a human–computer interaction task in a simulated manned/unmanned aerial vehicle cooperative mission. Experiments were set at three task difficulties: low, medium, and high, and were matched to a set time pressure or no time pressure for two seconds for low difficulty, three seconds for medium difficulty, and four seconds for high difficulty. The results showed that the touchscreens improved the participants’ response speed at a time pressure of 2 s or less compared with the use of a general input device; however, the task error rate also increased significantly. The higher the task difficulty was, the worse the performance was and the greater the perceived workload of the participants. The application of touchscreens in dynamic environments subjected the participants to greater physical demands. The performance of participants using a keyboard was no better than that when touchscreens were used during the experiment. Moreover, touchscreens did not significantly improve participant performance. The results support the possibility of using touchscreens in manned/unmanned aerial vehicle cooperative missions.

## 1. Introduction

Unmanned aerial vehicles (UAVs) are used in various applications (e.g., agriculture, mining, transportation, and ecological protection) because of their excellent reconnaissance and survival capabilities [1,2,3,4]. Today, the use of UAVs in manned/UAV (MAV/UAV) cooperative systems expands the capabilities and horizons of the user, but the ongoing effort to keep the system operational may pose new problems for war fighters [5,6,7]. Studies have been conducted on the factors influencing traditional human–computer interaction (HCI) tasks in flight scenarios. For example, using the human–computer interaction task of a button device, researchers examined the effect of error information alert designs on pilot performance [8]. In addition, human–computer interaction experiments with blind people showed that the error rate of key-press devices is lower [9]. However, little is known about the factors and mechanisms that affect MAV/UAV cooperative HCI tasks [10,11].

Touchscreens are widely used in daily life, such as in banking, retail, and mobile terminals [12,13]. Their applications are also being highlighted in in-vehicle and on-board devices because of their interactive nature [14,15,16]. The process of designing and improving touchscreens for in-vehicle applications has been a long-term and ongoing process [17,18,19]. The layout of touchscreens as a human–computer interaction interface in cockpits has been experimentally studied [20]. For example, the position of the screen can have an impact on pilot performance [21]. In addition, it has been reported that the application of touchscreens in the cockpit of an aircraft requires higher space for the upper limb movement of pilots [22]. In contrast, studies on the application of touchscreens in flight environments, particularly in MAV/UAV cooperative mission flight cockpits, and their impact on pilots, remain few. In an MAV/UAV cooperative flight mission, the pilot must perform both piloting and UAV surveillance tasks [23]. Large maneuvers and emergency scenarios are both mentally and physically demanding for pilots [24]. Touchscreens require pilots to respond in a new way because of their touch characteristics, in which a mistouch or error could cause an accident. Therefore, it is important to study the impact of touchscreen applications on pilots in MAV/UAV cooperative flight missions.

Research on the human impact of touchscreen devices in aircraft cockpits has attracted considerable interest. In commercial aircraft cockpits, touch interaction has been reported to be effective only in low-precision tasks [25]. Some studies have concluded that touchscreen panels can significantly reduce the pilot’s workload [26]. However, the importance of human-factor-engineering analysis has been emphasized [20,27]. The effects of touchscreen devices on reducing pilot workload in tactical flight situations are not obvious [28]. Current research shows that the target size, position, and vibration environment affect touch accuracy and precision [29,30]. However, little remains known about the effects of touchscreen applications when pilots are under time pressure in MAV/UAV cooperative missions.

Time pressure has been reported to affect pilot decision making. In particular, time pressure can increase the rate of poor decision making by less experienced pilots [31]. Some studies have suggested a relationship between time pressure and pilot workload [32]. Early studies confirmed that time pressure is related to the task structure and influences human decision making [33]. Time pressure can also cause a person’s mood to turn negative [34]. During pilot training, stress perception interacts with other factors that affect the efficiency of the pilot’s interaction [35]. It has been shown that, in an MAV/UAV cooperative mission, the pilot’s attention is biased toward UAV surveillance missions—a phenomenon that significantly increases with mission complexity [36]. Changes in task difficulty have been shown to cause changes in pilot electrocardiograph and electroencephalography signals, as well as in subjective evaluations [37]. Both time pressure and task difficulty significantly influence satisfaction with a search task strategy [38], with participants preferring simple textual information under time pressure [39]. In this study, the time pressure and task difficulty are considered to be environmental factors that influence pilot behavior in an emergency situation to explore the impact of touchscreens on pilot performance and workload in an MAV/UAV cooperative flight emergency situation.

In short, the purpose of this study is to analyze the impact of touchscreen devices on pilot performance and workload in an MAV/UAV cooperative flight mission to determine the feasibility of their application. A series of experiments were conducted on the application of touchscreens in a simulated MAV/UAV cooperative mission under different task difficulties, with and without time-pressure conditions. Common input devices (keyboards) were used as the controls to investigate the factors influencing touchscreen applications and the applicability of touchscreens.

## 2. Materials and Methods

### 2.1. Participants

Thirty undergraduate and graduate students (mean age = 23.0 years, SD = 2.3 years; 14 females and 16 males) from the School of Aeronautics and Astronautics of Northwestern Polytechnical University, Shaanxi, China with some knowledge of aviation participated in the experiment. Before the experiment, all participants were informed of the instructions and procedures, and they were asked to sign a written informed consent form. All participants had normal or corrected visual acuity, no color blindness or color weakness, and no cognitive impairment. The participants were requested to sleep sufficiently 24 h before the experiment. This study was approved by the Institutional Review Board of Northwestern Polytechnical University.

### 2.2. Experimental Design

**Figure 1:**
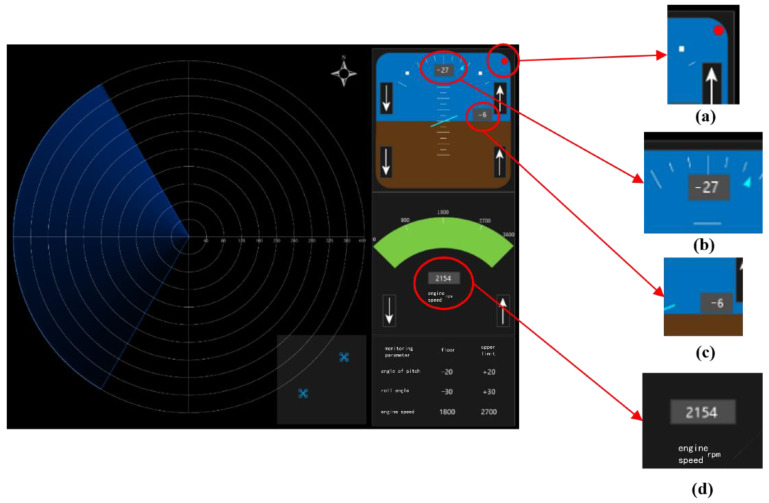
Simulated flight-parameter-monitoring interface: (**a**) prompt to adjust the pitch angle alarm; (**b**) pitch angle parameter; (**c**) roll angle parameter; and (**d**) engine speed parameter.

**Figure 2:**
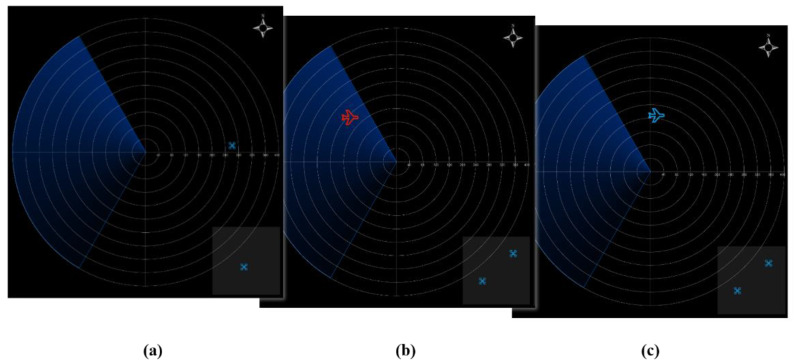
Simulated radar-scanning interface and UAV monitoring interface with (**a**) the appearance of enemy aircraft, (**b**) appearance of our own aircraft, and (**c**) UAV flying out of the designated box range.

An in-group three-way repeated measure design was used in the experiment. The experiment simulated the tasks of a pilot monitoring the flight parameters and flight range of two UAVs while searching for and identifying targets on the radar interface in an aircraft under cruise conditions. Independent variables included task difficulty (low, medium, and high), time pressure (2 s, 3 s, and 4 s, respectively, for low-, medium-, and high-difficulty tasks), and the use of a touchscreen or keyboard. A scenario with a low task difficulty had an alarm (red alarm light) and an action prompt (red alarm light displayed above the arrow where the action must be performed) (Figure 1a). A medium task difficulty was defined as a scenario with an alarm, but no action prompt, and a high task difficulty was defined as a scenario without an alarm or action prompt. In order to make the operator feel time constrained, time pressure was set based on the average reaction time of the operator before the experiment. The flight-parameter-monitoring task required participants to observe the aircraft parameters on the interface and maintain the pitch angle between −20° and 20°, roll angle between −30° and 30°, and engine speed between 1800 and 2700. When a parameter fell outside the specified range, the participant had to adjust the value to bring it back within range. The UAV monitoring task required the participant to observe the flight position of the UAV. If the UAV flew out of the box at the bottom-right corner of the monitoring interface, the participant had to click on the UAV icon to return it (Figure 2a). The target monitoring and identification tasks required the participant to observe the radar-scanning interface: the red aircraft icon was the enemy aircraft (Figure 2b), while the blue aircraft icon was their own aircraft (Figure 2c).

### 2.3. Procedures

Before the experiment, each participant was given a written informed consent form and a demographic information form, which they signed after learning about the experimental procedure. The experiment consisted of two phases: a 5 min practice phase and a 12 trial test phase (with/without time pressure × with/without a touchscreen × with three task difficulty levels). Each experimental phase lasted 10 min. The participants in each experiment were asked to observe the simulated parameter display interface. The radar sweep area was on the left, while the pitch, roll, and engine speed parameters were displayed and adjusted from top to bottom on the right. The participants were asked to monitor the above parameters and keep them within the normal range, and to adjust them by clicking on the corresponding marks on the screen with the mouse (for non-touchscreen experimental conditions) or a finger (for touchscreen experimental conditions). Parameters that exceeded the normal range had to be adjusted downward, while those that fell below the normal range had to be adjusted upward. The participants also observed the target on the left side of the radar sweep screen. The red aircraft had eliminated by clicking it with the mouse or a finger (under touchscreen conditions). When the UAV flew out of the square area on the lower-right side, it had to be returned by clicking it with the mouse or a finger (under touchscreen conditions). Under the time-pressure condition, different time pressures (2 s, 3 s, 4 s for low-, medium-, and high-difficulty tasks, respectively) were matched according to task difficulty. The participants’ incorrect and missed operations were recorded by the experimental program. At the end of the experiment, the participants filled out the NASA-TLX questionnaire [40,41,42]. The entire experiment was completed within 1.5 h. The ambient temperature for the experiment was maintained at 23 °C.

### 2.4. Dependent Variables

The dependent variables include the task performance (completion time and error rate) and the subjective workload ratings. The completion time refers to the time between the appearance of an abnormality and the participants’ response. The correctness rate refers to the percentage of correct actions of the participant in all operations (omissions, misjudgments, and incorrect execution are all considered errors). The perceived workload was measured using a modified NASA-TLX scale [41,43,44,45,46] with six bipolar dimensions: mental demand (MD); physical demand (PD); time demand (TD), level of effort; level of performance; and level of frustration. The first three dimensions (MD, PD, and TD) reflected the task-related characteristics, such as task complexity. The modified NASA-TLX had values ranging from 0 to 10, in which a higher value indicated a greater task load.

### 2.5. Data Analysis

The participants’ performance (task completion time, error rate) and workload (NASA-TLX) data were collected under different experimental conditions, i.e., time pressure, screen type, and task difficulty. The data were statistically analyzed using SPSS (version 26.0, Chicago, IL, USA). Repeated measurements of the analysis of variance (ANOVA) were used to assess the effects of time pressure, screen type, and task difficulty on task performance. A three-way ANOVA was used to assess the effects of time pressure, screen type, and task difficulty on the workload. Bonferroni post-hoc comparison tests were used to evaluate the differences among the partial pair-wise means (α=0.05).

## 3. Results

### 3.1. Task Completion Time

**Figure 3:**
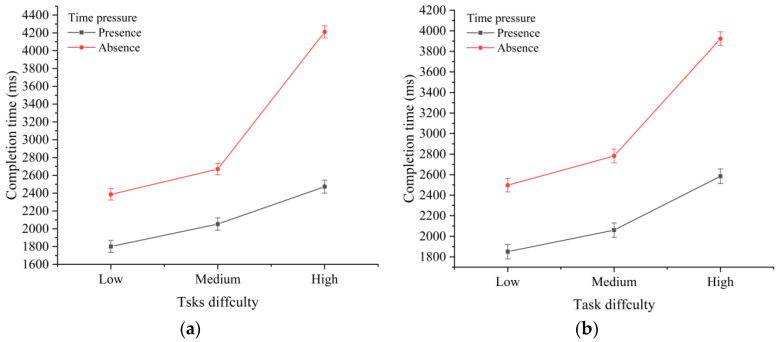
Effects of task difficulty and time pressure on the response time under (**a**) touchscreen and (**b**) normal screen conditions.

Table 1 presents the results of the descriptive and ANOVA analyses of the task completion times under different time pressures and task difficulties. The results show that, in addition to the screen type (F(1,846)=0.185,p=0.667), the task completion time was significantly affected by the task difficulty (F(2,846)=314.242,p<0.01) and time pressure (F(1,846)=570.229,p<0.01). We also found a significant interaction effect of the task difficulty and time pressure on the task completion time (F(2,1023)=56.135,p<0.01). However, there were no significant interaction effects between the screen type and time pressure (F(1,8462)=0.967,p=0.325), and between the screen type and task difficulty (F(2,8462)=1.775,p=0.17), on the completion time. Figure 3 shows the interaction effect of the time pressure and task difficulty on the task completion time under (a) general screen (input device was the keyboard) (F(1,853)=246.80,p<0.01) and (b) touchscreen (F(1,751)=310.03,p<0.01) conditions. There was a significant interaction effect between the time pressure, screen type, and task difficulty (F(2,8462)=4.12,p<0.05) on the completion time.

**Figure 4:**
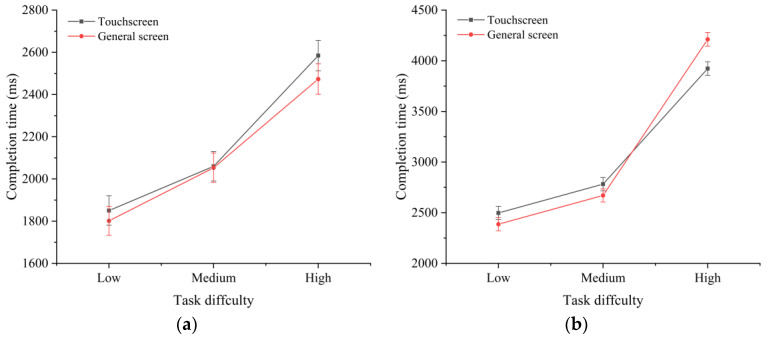
Interaction effects between the screen type and task difficulty (**a**) with and (**b**) without time-pressure conditions on the completion time.

**Figure 5:**
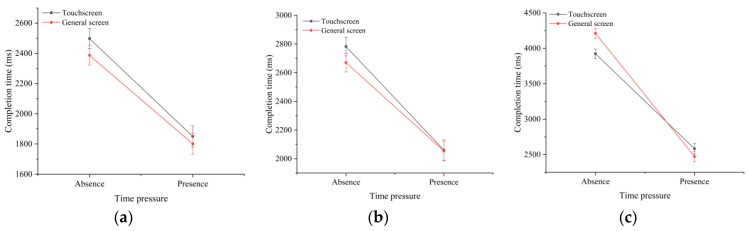
Interaction effects of the screen type and time pressure on the completion time of tasks with different difficulties: (**a**) low difficulty; (**b**) medium difficulty; and (**c**) high difficulty.

As shown in Figure 4, further analysis revealed a significant interaction effect between the screen type and task difficulty in the absence (F(2,8462)=194.39,p<0.01) and presence (F(2,8462)=506.17,p<0.01) of time pressure. The screen type and time pressure had a significant interaction effect on the completion time of high-difficulty tasks (F(2,3060)=5.36,p<0.01), but there were no significant interaction effects with other task difficulties. These effects are shown in Figure 5.

### 3.2. Error Rate

**Figure 6:**
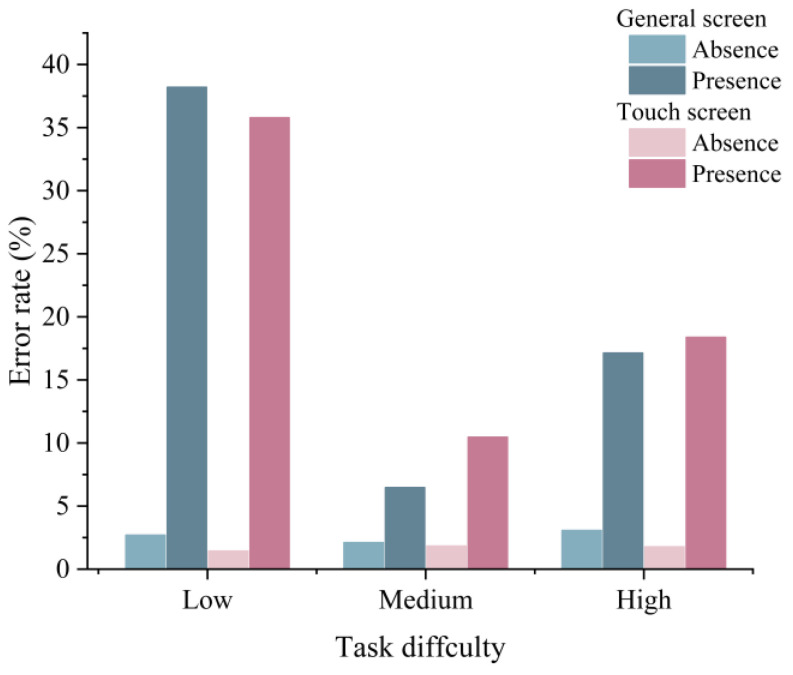
Error rate under different time-pressure, screen-type, and task-difficulty conditions.

The time pressure was found to have a significant effect on the error rate (F(1,10)=12.485,p<0.01). In contrast, neither the screen type (F(1,10)=0.025,p=0.87) nor the task difficulty (F(2,9)=1.262,p=0.33) affected the error rate. Only one error rate was obtained under each experimental condition. The analysis of error rates using the coefficient of variation (CV) showed significant differences at different time pressures (C.V.=0.1514>15%). However, the differences were not significant in the remaining cases. Figure 6 shows the error rates under different experimental conditions.

**Figure 7:**
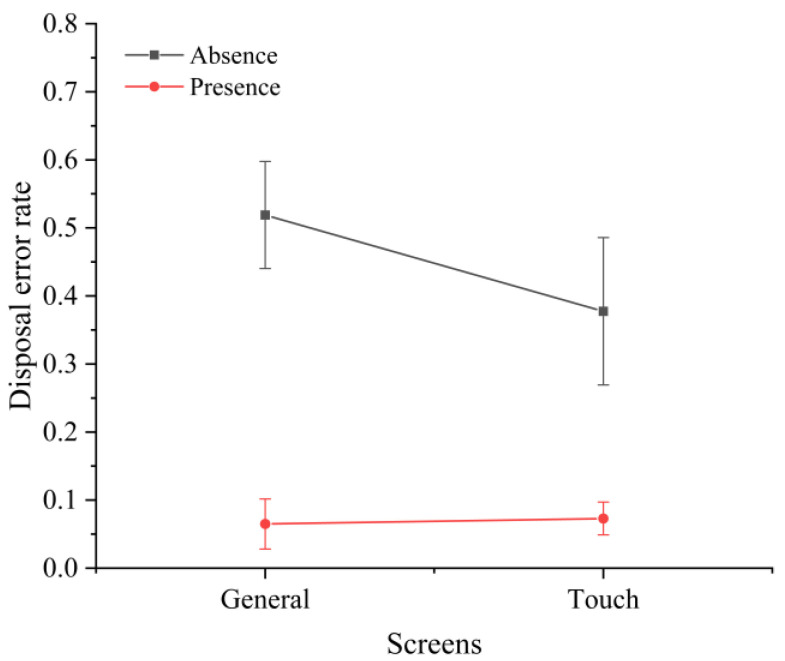
Interaction effect between screen type and time pressure on the disposition error rate.

**Figure 8:**
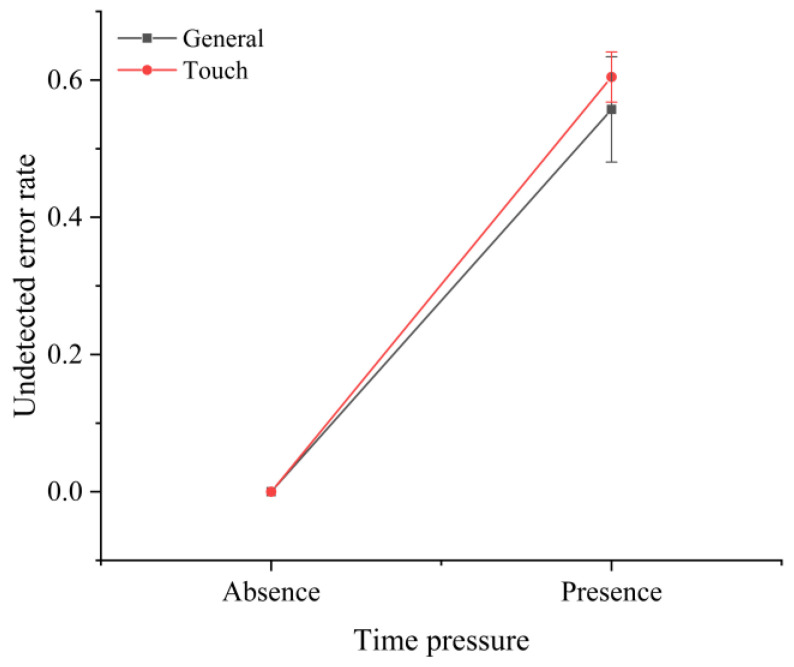
Effect of time pressure on undetected errors.

**Figure 9:**
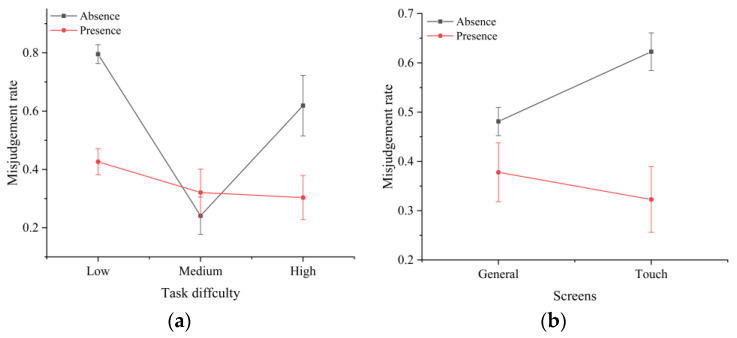
Interaction effects between the (**a**) task difficulty and time pressure and (**b**) screen type and time pressure on misjudgment.

The analysis of various error types showed that the screen type and time pressure significantly affected the disposition errors (F(2,30)=2.326,p<0.001). This was demonstrated by the fact that the use of touchscreens in the absence of time pressure reduced the percentage of disposition errors among all error types (Figure 7). It is worth emphasizing that time pressure had a significant effect on the ratio of undetected errors to all error types. Without time pressure, there were no undetected errors; with time pressure, the ratio of undetected errors to all error types increased to over 50% (Figure 8). In addition, there was a significant interaction effect between the task difficulty and time pressure (F(1,30)=3.223,p<0.001) (Figure 9a), and between the screen type and time pressure (Figure 9b) (F(1,30)=2.592,p<0.001) on the misclassification errors.

### 3.3. Perceived Workload

The time pressure significantly affected the overall workload (F(1,358)=42.977,p<0.001) and the six subdimensions of the workload: MD (F(1,358)=21.241,p<0.001); PD (F(1,358)=10.128,p=0.002); TD (F(1,358)=134.891,p<0.001); performance (F(1,358)=6.570,p=0.011); effort (F(1,358)=9.107,p=0.003); and frustration (F(1,358)=38.614,p<0.001). The task difficulty affected the overall workload (F(2,357)=19.208,p<0.001); performance (F(2,348)=0.795,p=0.452); MD (F(1,358)=63.067,p<0.001); PD (F(1,358)=12.773,p<0.001); TD (F(1,358)=3.772,p=0.024); effort (F(1,358)=5.891,p=0.003); and frustration (F(1,358)=8.352,p<0.001). The screen type only had a significant effect on the PD (F(1,348)=4.900,p=0.028). Table 2 shows the perceived workload average statistics for the different conditions.

**Figure 10:**
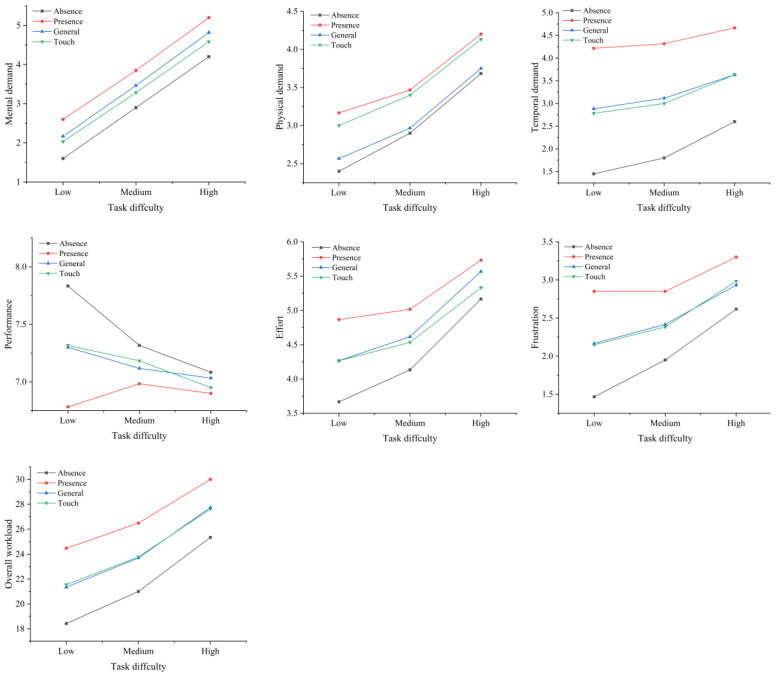
Effects of time pressure, screen type, and task difficulty on the perceived workload and its six subdimensions.

There were no significant interaction effects between the time pressure and screen type (F(1,348)=0.024,p=0.877), between the time pressure and task difficulty (F(2,348)=0.272,p=0.762), and between the screen type and task difficulty (F(2,348)=0.014,p=0.986) on the perceived workload and its six subdimensions. In addition, there were no significant interaction effects between the time pressure, screen type, and task difficulty on the perceived workload and its subdimensions (F(2,348)=0.056,p=0.946). Figure 10 shows the effects of the time pressure, screen type, and task difficulty on the perceived workload and its subdimensions.

## 4. Discussion

This study explores the applicability of touchscreens to HCI tasks in typical MAV/UAV cooperative missions. Control experiments with a normal input device showed that touchscreens were more physically demanding than the normal device, but there were no significant differences in the task performance and the perceived workload. Specifically, in MAV/UAV cooperative missions, variations in the time pressure and mission difficulty significantly affected the mission completion time. However, only the time pressure significantly affected the task error rate with touchscreen use. The perceived workload of the participants in the touchscreen application scenario was significantly influenced by the time pressure and task difficulty. The task performance and perceived workload when using touchscreens and other input devices were not significantly different, indicating that touchscreens were equally suitable for MAV/UAV cooperative missions as other input devices.

### 4.1. Time Pressure

The time pressure significantly influenced the decision-making and cognitive process of the operator, leading to poor operational performance: this is consistent with the results of previous studies [31,47,48]. This was demonstrated in experiments that used both touchscreens and keyboard. There was no significant difference between the results of the two comparison tests. One possible reason for this is that the time pressure limits the amount of visual information received by the participants, which reduces the accuracy of the acquired information, leading to poor decision making [49,50]. It is worth noting that the presence of time pressure significantly reduced the time that the participants spent performing a task; however, the corresponding error rate significantly increased. Using a touchscreen slightly reduced the participants’ response time under time pressure compared to the use of a general input device. However, this does not indicate a significant performance improvement. This phenomenon has been reported in studies on the effect of time pressure on HCI task performance [51] with potentially effective solutions, such as the use of assisted decision-making systems [52]. We found that time pressure significantly increased the error rates in all three levels of task difficulty, with or without the use of touchscreens. The largest error rate increase of 35% was observed in the low-difficulty tasks. This may be attributed to the negative effect of time pressure on participants’ performance. Time pressure had a greater effect on participant performance than task difficulty [51,53]. Because low-difficulty tasks provide detailed operational cues (instructions on how to handle errors) and the corresponding time pressure is highest for low-difficulty tasks, we hypothesized that providing detailed information under high time pressure would increase error rates, and that a detailed information display is not always an efficient information delivery feature.

Interestingly, the presence of time pressure significantly reduced the proportion of disposition and miscalculation errors among all error types, but significantly increased the proportion of undetected errors. Compared to keyboards, the use of touchscreens resulted in fewer misjudgment errors under time pressure; however, there was no significant change in the overall error rate. This reflects the participants’ method of dealing with time pressure, focusing their time and attention on the first errors they find. When performing a task, touchscreens may be more immersive than keyboards. Participants under time pressure may forget important informational premises and have a poor working memory, which impairs their capacity for reason and judgment [54,55]. Our data from the NASA-TLX scale showed that the presence of time pressure significantly increased the participants’ perceived workload, which is consistent with previous studies [56,57]. Time pressure is a double-edged sword [58] that can reduce the operator completion time but can also negatively impact perceived workload and error rates. Setting the appropriate time constraints could result in new discoveries and implications for pilot training and flight safety. Touchscreen applications can place high demands on the physical strength of the participant, which is amplified by the time pressure. When touchscreens are applied to HCI tasks in an MAV/UAV cooperative mission, efforts should be made to reduce the time pressure for improved performance.

### 4.2. Task Difficulty

The task completion time of the participants using a touchscreen increased with the difficulty of the task, while the lowest error rate was obtained for medium-difficulty tasks. Several studies have applied tasks of varying difficulties to explore participants’ performance and workload [59,60]. Compared with the use of ordinary input devices, the use of touchscreens led to a slight increase in the response rate at a time pressure of 4 s, as well as a reduction in the misjudgment error rate. In brief, the participants’ perceived workload increased with the task difficulty; however, under time pressure, the participants’ performance demands were at their worst level with medium-difficulty tasks. This may be attributed the fact that some participants clearly perceived the shift from low- to medium-difficulty tasks, and this effect was amplified by the time pressure. These phenomena provide suggestions on the potential applications of touchscreens in typical MAV/UAV cooperative missions. Setting different time pressures for different task difficulties could be helpful when using touchscreens in MAV/UAV cooperative missions.

The effects of the time pressure and task difficulty on HCI task performance and perceived workload in an MAV/UAV cooperative mission did not differ significantly between touchscreens and normal input devices, which is partially consistent with previous studies [61,62]. The use of touchscreens made participants more manipulative, as shown by the results of this study. The deployment of touchscreens slightly improved participants’ completion time under the conditions of high-difficulty tasks and time pressure. In the absence of time pressure, the touchscreen mistake rate was marginally lower than that of a standard device. One possible reason for this is that touchscreens provide participants with a greater and more direct sense of manipulation of the target [63]. The use of touchscreens also increased the perceived physical workload, possibly because of the increased amount and range of motion of the participants’ upper extremities. Research has been conducted on the posture and physical demands of touchscreen use [30,64,65]. The results of the current study verified, to some extent, that touchscreens can be used in HCI tasks in MAV/UAV cooperative missions. Aircraft pilot system designers should consider the impact of time pressure and task difficulty on touchscreen applications.

### 4.3. Limitation

The present study has several limitations that could be addressed by future work. First, the way in which manned and unmanned aircraft information is displayed, such as the way the information is laid out, differences in alert messages, and the degree of information integration, may have an impact on pilot performance [48,66,67,68], which was not examined in this study, but deserves further exploration. Second, we used pilot performance (completion time and error rate) and NASA-TLX measurements to comprehensively evaluate the use of the touchscreen. However, the analysis of physiological measurements, such as eye movements, electroencephalogram (EEG), and other physiological data, was not conducted in the study [69,70], and future studies could carry out measurements of physiological data to make the results more comprehensive. Third, our study only examined the application of touchscreen and keypad devices in a simple simulated flight environment. Future studies can include environmental disturbances, such as vibration and noise, to make the results more applicable in specific environments.

## 5. Conclusions

This study investigated the applicability of touchscreens to HCI tasks in MAV/UAV cooperative missions, and the effects of time pressure and task difficulty variations. The existence of time pressure and increased task difficulty worsened the performance of the participants in touchscreen applications. The perceived workload of the participants also increased. Controlled experiments with a keyboard showed that the task completion time was shorter when touchscreens were used under time pressure in high-difficulty tasks; however, the error rates were not significantly different in the two cases. Therefore, incorporating assisted decision-making in the system and rational workload distribution are feasible proposals in an MAV/UAV cooperative mission. In surveillance-oriented MAV/UAV cooperative missions, the keyboard did not demonstrate advantages over touchscreens in terms of the participants’ performance and perceived workload. However, there was also no significant improvement in participants’ performance and perceived workload with the use of touchscreens. Therefore, the preliminary findings do not reject the use of touchscreens for HCI missions in MAV/UAV cooperative missions.

## Figures and Tables

**Table 1:** Completion time (milliseconds (ms)) statistics under different time pressures and task difficulties.

	Touchscreen		General Screen	
	Mean (SD)	F Value (*p* Value)	Mean (SD)	F Value (*p* Value)
Time pressure		310.03 (<0.05)		246.80 (<0.05)
Presence	2156.02 (628.47)		2098.94 (628.01)	
Absence	3055.47 (2211.58)		3052.62 (2797.55)	
Task difficulty		172.52 (<0.01)		185.91 (<0.01)
Low	2191.86 (948.48)		2114.09 (1807.25)	
Medium	2443.47 (1326.35)		2383.36 (1391.10)	
High	3299.04 (2404.99)		3394.61 (2823.41)	

**Table 2:** Mean (SD) of the perceived workload by time pressure, screen type, and task difficulty.

	Mental Demand (MD)	Physical Demand (PD)	Temporal Demand (TD)	Performance (P)	Effort (E)	Frustration (F)	Overall (OA)
Time pressure							
Absence	2.90 (1.98)	2.99 (1.80)	1.95 (1.71)	7.41 (2.10)	4.32 (2.95)	2.01 (1.28)	21.58 (10.54)
Presence	3.88 (2.07)	3.61 (1.87)	4.40 (2.26)	6.89 (1.75)	5.21 (2.60)	3.00 (1.71)	26.99 (12.26)
Screen							
General screen	3.48 (2.06)	3.09 (1.73)	3.21 (2.33)	7.15 (1.94)	4.82 (2.85)	2.51 (1.45)	24.26 (12.36)
Touchscreen	3.30 (2.10)	3.51 (1.97)	3.14 (2.36)	7.15 (1.96)	4.71 (2.77)	2.51 (1.72)	24.32 (12.88)
Task difficulty							
Low	2.10 (1.66)	2.78 (1.87)	2.83 (2.42)	7.31 (2.20)	4.27 (2.92)	2.16 (1.59)	21.45 (12.66)
Medium	3.38 (1.69)	3.18 (1.74)	3.06 (2.25)	7.15 (1.87)	4.58 (2.68)	2.40 (1.49)	23.75 (11.72)
High	4.70 (2.01)	3.94 (1.80)	3.63 (2.30)	6.99 (1.74)	5.45 (2.70)	2.96 (1.59)	27.67 (12.14)

## Data Availability

The data are partially restricted because they contain some of the participants’ private data.
